# COMMUNITY ENGAGEMENT, SOCIAL SUPPORT, AND RELATIONSHIP DYNAMICS AMONG OLDER ADULTS POST-COVID-19

**DOI:** 10.1093/geroni/igae098.3830

**Published:** 2024-12-31

**Authors:** Limei Chen, Mengzhao Yan, Philip Rozario

**Affiliations:** George Mason University, Fairfax, Virginia, United States; University of Southern California, Los Angeles, California, United States; Adelphi University School of Social Work, Adelphi University School of Social Work, New York, United States

## Abstract

Social integration theory and social capital theory suggest the positive effect of community participation and the benefits of social connections gained in the community on health and wellbeing in later life. However, it is unclear whether these positive effects persist under the social restrictions imposed by the COVID-19 pandemic. To understand if the benefits of community participation can withstand such unprecedented disruptions, this study explores how community participation influences spousal/partner relationships, physical health, and mental health among older adults before and after COVID-19. Using linked data of the National Social Life, Health, and Aging Project (NSHAP) Round 3 and Covid-19 Study, we assess the impacts of volunteering, attending meetings, and religious activities using multinomial regrenssion models. Findings reveal that community participation, particularly volunteering and attending meetings, is strongly associated with improved relationship outcomes, even under stress from events related to the COVID-19 pandemic. Religious activities also support better relationships, especially when stressful events are present. While social support further enhances these positive effects, it can diminish the independent benefits of volunteering. For physical and mental health, community engagement serves as a protective factor, mitigating the adverse effects of stressful events. These results underscore the crucial role of active community involvement in promoting well-being and maintaining positive spousal/ partner relationship dynamics among older adults, highlighting its importance as a buffer against stressful events.

